# Frequency of SARS-CoV-2 Infections among Healthcare Workers in Germany: 3-Year Follow-Up Study

**DOI:** 10.3390/idr16040047

**Published:** 2024-07-19

**Authors:** Christian Stammkötter, Laura Thümmler, Johannes Korth, Beate Marenbach, Peer Braß, Peter A. Horn, Monika Lindemann, Ulf Dittmer, Oliver Witzke, Hana Rohn, Adalbert Krawczyk

**Affiliations:** 1 Department of Infectious Diseases, West German Centre of Infectious Diseases, University Medicine Essen, University Hospital Essen, University Duisburg-Essen, 45147 Essen, Germany; cstammkoetter@gelsennet.de (C.S.); laura.thuemmler@uk-essen.de (L.T.); peer.brass@uk-essen.de (P.B.); oliver.witzke@uk-essen.de (O.W.); hana.rohn@uk-essen.de (H.R.); 2 Institute for Transfusion Medicine, University Hospital Essen, University Duisburg-Essen, 45147 Essen, Germany; beate.marenbach@uk-essen.de (B.M.); peter.horn@uk-essen.de (P.A.H.); monika.lindemann@uk-essen.de (M.L.); 3 Department of Nephrology, University Medicine Essen, University Hospital Essen, University Duisburg-Essen, 45147 Essen, Germany; korth@dialyse-kortumpark.de; 4 Practice for Kidney Diseases, Dialysis and Apheresis, 44789 Bochum, Germany; 5 Institute for Virology, University Hospital Essen, University Duisburg-Essen, 45147 Essen, Germany; ulf.dittmer@uk-essen.de

**Keywords:** SARS-CoV-2, healthcare workers, exposure risk

## Abstract

The emergence of SARS-CoV-2 in 2019 led to a global pandemic with a significant impact on healthcare systems. Healthcare workers were particularly vulnerable due to frequent contact with COVID-19 patients. Despite vaccination, they remained at higher risk as the vaccines provided limited protection against infection with viral variants, like Delta or Omicron BA.1 and BA.5. Three years after the onset of the pandemic, we evaluated SARS-CoV-2 infection frequencies among healthcare workers with varying levels of patient contact: high-risk (frequent COVID-19 patient contact), intermediate-risk (non-COVID-19 patient contact), and low-risk (no patient contact). We assessed their cellular and humoral immune responses based on their vaccination status and number of prior infections. SARS-CoV-2-specific antibodies were measured by immunoglobulin ELISA, and neutralizing antibody titers were determined against the viral variants D614G, Delta, and Omicron BA.1 and BA.5. Cellular immune responses were analyzed using an interferon-γ ELISpot. Notably, three years into the pandemic, healthcare workers in daily contact with COVID-19 patients did not have higher infection rates compared to healthcare workers with non-COVID-19 patient contact or no patient contact. Immune responses were similar across all groups, highlighting the effectiveness of vaccination and current hygiene standards in preventing virus transmission from patients to staff.

## 1. Introduction

The severe acute respiratory syndrome corona virus type 2 (SARS-CoV-2) emerged in 2019 and caused a still ongoing pandemic. An infection with SARS-CoV-2 can be asymptomatic, mild/moderate, severe, or critical. The symptoms range from mild pulmonary manifestations to fatal pneumonia and respiratory distress syndromes [1]. In a mild case, a SARS-CoV-2 infection is almost indistinguishable from influenza or common cold, but in some cases, there are specific symptoms such as loss/change in smell/taste perception, which may also persist after acute disease; these are more likely in cases of severe COVID-19 [2,3]. Notably, loss of smell/taste was associated with the D614G mutation, which was predominantly observed during the early stage of the pandemic, but less frequently observed for newly emerging variants such as Omicron [4]. 

Individuals with certain pre-existing conditions such as immunosuppression, cancer, chronic obstructive pulmonary disease (COPD), and other risk factors have a higher risk of developing a severe course of infection [5]. Healthcare workers (HCW) with contact with COVID-19 patients were discussed to be at an increased risk for being infected with SARS-CoV-2 [6]. Early vaccination campaigns for HCW and infection prevention measures during the beginning of the pandemic resulted in a low infection rate even in HCW with frequent contact with COVID-19 patients at one year after the outbreak of COVID-19 in Germany [7]. The seroprevalence in HCW was reported at 1.6% in March 2020 and 5.1% at the end of 2020 [8]. Other studies reported that HCW with high levels of patient contact exhibited a greater likelihood of infection both before and after the emergence of the Omicron variant [9]. The emergence of SARS-CoV-2 variants such as Delta or Omicron BA.1 and BA.5 led to an increasing number of vaccine breakthrough infections in 2022 and 2023 [10,11]. As Omicron spread and community health measures relaxed, SARS-CoV-2 infections surged even among highly vaccinated healthcare workers. These infections, though mostly mild, occurred primarily outside hospitals, since protective measures remained enforced across healthcare premises. Pre-existing immunity prevented severe COVID-19 but not mild infections due to Omicron’s immune evasion. Non-pharmaceutical interventions in hospitals effectively prevented SARS-CoV-2 transmission [9]. Until today, roughly 50% of the German civil population recovered from COVID-19 at least once, and the infection rates can be assumed to be much higher [12]. However, it remains unclear to what extent HCW with frequent contact with COVID-19 patients are more likely to be infected with SARS-CoV-2 than HCW with less or no work-related contact with the respective patients over a longer period of time. 

In the present study, we investigated the incidence of SARS-CoV-2 infections among HCW with frequent contact with COVID-19 patients (high-risk group), employees with contact with non-COVID-19 patients (intermediate-risk group), and employees without any contact with patients (low-risk group) as a follow up study from our hospital three years after the onset of the pandemic. Furthermore, we evaluated humoral and cellular immune responses, which represent the protective immunity against SARS-CoV-2 [13,14]. Moreover, we investigated the relationship between the level of the humoral and cellular immune responses and the number of infections or breakthrough infections that can boost the immune system [15]. The data thus obtained provide an important insight into whether HCW with frequent contact with COVID-19 patients were at increased risk of SARS-CoV-2 infection over three years of the pandemic, especially with newly emerging variants [13,14,15]. 

## 2. Materials and Methods

### 2.1. Study Design

In total, we enrolled 73 healthcare workers from the University Hospital of Essen, spanning the period from 12 March to 8 May 2023—over three years since the onset of the pandemic. All HCW were interviewed to determine the number of prior SARS-CoV-2 vaccinations, infections/breakthrough infections, and demographic data such as gender and age. HCW were classified into three distinct risk groups according to their frequency of contact with COVID-19 patients. HCW with daily exposure to known or suspected SARS-CoV-2 positive patients were assigned to the high-risk group [7]. The intermediate-risk group included those with daily contact with non-COVID-19 patients, and the low-risk group comprised HCWs without patient contact. To ensure safe patient care in designated COVID-19 wards (high-risk areas), a comprehensive local hygiene protocol was established at our hospital. This protocol mandated the use of personal protective equipment (PPE), which included respiratory protection in the form of FFP-2 respirators, disposable coats, waterproof gowns, single-use gloves, and face shields. For staff working inwards without known or suspected COVID-19 patients (classified as intermediate-risk areas), adherence to basic hygiene standards as outlined by the World Health Organization (WHO) was required. Additionally, all staff members across the hospital were required to wear FFP-2 respirators by the end of 8 April 2023, regardless of their assigned area. Healthcare workers who have contact with COVID-19 patients continue to wear appropriate personal protective equipment, including FFP2 masks. Furthermore, all hospital personnel were obligated to strictly follow hand hygiene practices in accordance with the WHO guidelines. These measures were implemented to minimize the risk of infection transmission and ensure the safety of both healthcare workers and patients throughout the facility. SARS-CoV-2 screening changed over the course of the pandemic. At the beginning of the pandemic, HCW with symptoms, as well as HCW who had direct contact with infected individuals, were tested. Until 8 March 2023, all HCW were required to test themselves twice a week, regardless of the presence of symptoms. After that, only symptomatic HCW were tested. The testing was carried out in the same way for all HCW, regardless of the area in which they worked.

### 2.2. ELISA

SARS-CoV-2-specific antibodies were detected using a CE-marked, quantitative anti SARS-CoV-2-IgG-ELISA (anti-SARS-CoV-2-QuantiVac-ELISA, Euroimmun, Lübeck, Germany) according to the manufacturer’s instructions. Results were given as BAU/mL. Semiquantitative ELISA kits (Anti-SARS-CoV-2-NCP-ELISA, Euroimmun, Lubeck, Germany) were used to determine IgG and IgM antibodies against SARS-CoV-2 nucleocapsid according to manufacturer’s instructions. The results were recorded as a sample/control ratio. A ratio of >1.1 was considered positive, a ratio of ≥0.8 to <1.1 was regarded as borderline, and a ratio of <0.8 was considered negative.

### 2.3. Neutralization Assay

To evaluate the neutralizing antibody titers of sera from healthcare workers, we employed a standard endpoint dilution assay as previously described [16,17]. The sera were serial diluted (1:20 to 1:2560) and incubated with 100 TCID_50_ of SARS-CoV-2 D614G (wild-type), Delta (B1.617.2), or Omicron BA.1 or BA.5 for one hour at 37 °C, 5% CO_2_. Afterward, the mixtures were added to confluent A549-AT cells in 96 well microtiter plates and incubated for three days at 37 °C and 5% CO_2_ [18]. Cell cultures were stained with crystal violet (Carl Roth, Karlsruhe, Germany) and examined for the presence of cytopathic effects (CPE) by light microscopy. The neutralizing titer was defined as the reciprocal of the highest serum dilution at which no CPE was observed [19,20].

### 2.4. In-House ELISpot Assay

The cellular immune response was analyzed by using an *in-house* interferon (IFN)-γ Enzyme-linked-immuno-Spot (ELISpot) assay as described previously [20]. Briefly, 250,000 peripheral mononuclear cells (PBMC) were incubated in the presence or absence of either PeptTivator^©^ wild-type protein S, S1 of Delta S.AY, S1 of Omicron BA.5 or S1 of Omicron BQ1.1 (600 pmol/mL of each peptide, Miltenyi Biotec, Bergisch Gladbach, Germany) in 150 µL of AIM-V^©^ for 18 h at 37 °C in ELISpot plates coated with 60 µL of monoclonal antibodies against IFN-γ (10 µg/mL of clone 1-D1K; Mabtech). After incubation, plates were washed and incubated for 1 h with 50 µL of the alkaline phosphatase-conjugated monoclonal antibody against IFN-γ (clone 7-B6–1, Mabtech), and diluted 1:200 with phosphate-buffered saline plus 0.5% bovine serum albumin. Afterward, plates were washed again, and 50 µL of nitro blue tetrazolium/5-bromo-4-chloro-3-indolyl-phosphate was added for 7 min until purple spots appeared. Spots were quantified with an ELISpot reader (AID Fluorospot, Autoimmun Diagnostika GmbH, Strassberg, Germany). Spots increment was determined as the stimulated minus non-stimulated values.

### 2.5. Ethics

The study was approved by the Ethics commission of the University of Essen (approval no. 20-9753-BO, approved on 16 December 2020) and was conducted according to the ethical standards of the 1964 Declaration of Helsinki and its subsequent amendments. All participants provided written informed consent before enrollment into the study.

### 2.6. Statistics

Statistical analysis was performed using GraphPad Prism 10.0 software (GraphPad, San Diego, CA, USA). Mann–Whitney U tests were used for numerical variables. *p*-values < 0.05 were considered significant.

## 3. Results

### 3.1. Study Overview

**Figure 1:**
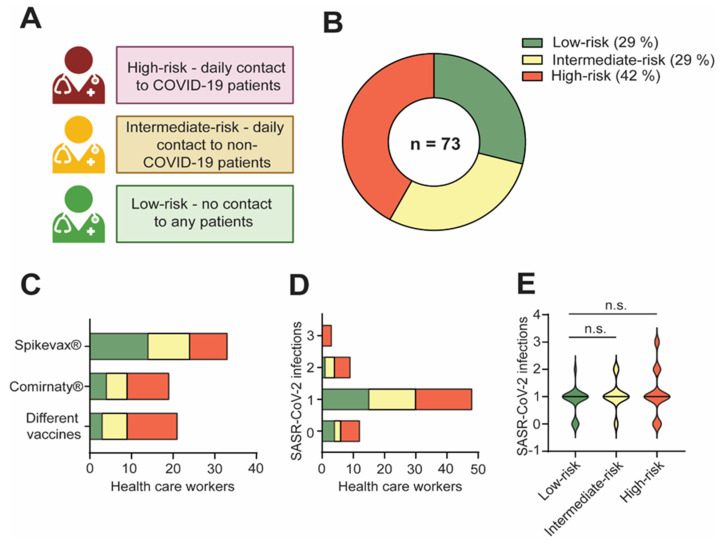
Characteristics of study cohort. (**A**) The cohort was categorized into three groups according to the frequency of their exposure to COVID-19 patients. This included a high-risk group comprising healthcare workers (HCW) with daily contact with COVID-19 patients, an intermediate-risk group consisting of HCW with daily contact with non-COVID-19 patients, and a low-risk group comprising HCW who had no contact with patients. (**B**) Distribution of HCW according to the risk classification. (**C**) Vaccination history of HCW within the three risk groups. (**D**) Frequency of SARS-CoV-2 infections across all risk groups. (**E**) Absolute numbers of reported SARS-CoV-2 infections based on exposure risk. Differences between groups were analyzed by Kruskal–Wallis test (n.s. = not significant).

The cohort of 73 healthcare workers was stratified into three categories based on their exposure frequency to COVID-19 patients (Table 1, Figure 1A). The low-risk group comprised 21 (28%) healthcare workers who had no patient contact, the intermediate-risk group comprised 21 (28%) HCW with daily contact with non-COVID-19 patients, and the high-risk group included 31 (44%) HCW who had daily contact with COVID-19 patients (Figure 1B). The low-risk group contained 5 men and 16 women with a median age of 34 (22–62) years. The intermediate-risk group contained 2 men and 19 women with a median age of 44 (23–62) years. The high-risk group consisted of 12 men and 19 women with a median age of 39 (23–70) years (Table 1).

The HCW were tested for humoral and cellular immune responses towards different SARS-CoV-2 variants. There was no significant difference between the groups in terms of the median age, but there was a difference in gender distribution. All HCW had been vaccinated three or four times. The last vaccination took place at a median of 486 (90–789) days before blood collection. The latest infection took place at a median of 274 (15–1186) days prior to blood collection.

Within the low-risk HCW, 14 received vaccinations exclusively with Spikevax^®^ (Moderna), 4 with Comirnaty^®^ (BioNTech/Pfizer), and 2 with both Spikevax^®^ and Comirnaty^®^. In the intermediate-risk group, 10 HCW were solely vaccinated with Spikevax, 5 with Comirnaty, and 6 with a combination of Spikevax and Comirnaty^®^. The high-risk group saw 9 HCW vaccinated with Spikevax^®^, 10 with Comirnaty^®^, 11 with both vaccines, and 1 individual received vaccinations with Comirnaty^®^ and Vaxzevria (AstraZeneca). No significant differences were observed in the number or type of vaccinations across groups (Figure 1C).

A total of 61 (84%) HCW reported at least one confirmed SARS-CoV-2 infection via PCR or rapid antigen test, while 12 (16%) HCW remained uninfected over three years since the pandemic’s commencement. Among those without SARS-CoV-2 infection, four were in the low-risk group, two in the intermediate-risk group, and six in the high-risk group. In the low-risk group, 15 HCW experienced one previous SARS-CoV-2 infection, and 1 person had two. In the intermediate-risk group, 15 HCW were infected once, and 4 twice. Among the high-risk group, 18 HCW had one infection, 5 were infected twice, and 2 reported three previous infections (Figure 1D). Notably, no significant differences emerged in the frequency of SARS-CoV-2 infections among the three risk groups (Figure 1E).

### 3.2. HCW Had Comparable Immune Responses Regardless of Contact with COVID-19 or Non-COVID-19 Patients

**Figure 2:**
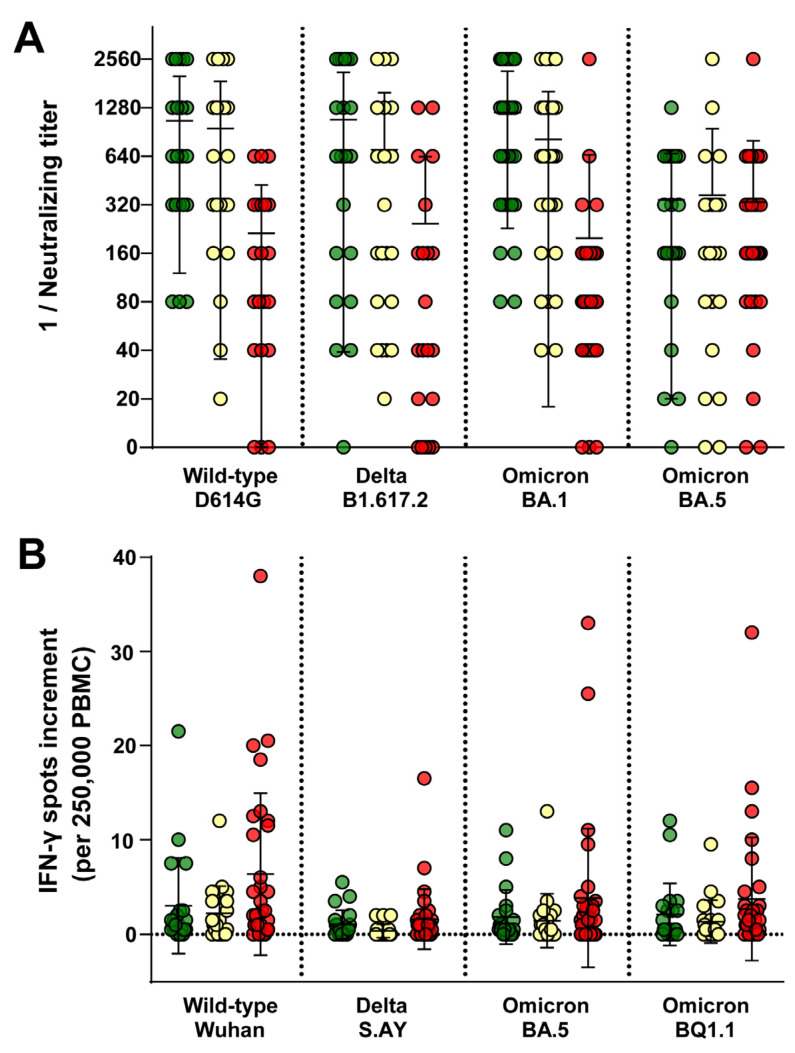
Humoral and cellular immunity against different SARS-CoV-2 variants. The neutralizing antibody titers and IFN-γ spot increments against different SARS-CoV-2 variants were compared among HCW of different risk of SARS-CoV-2 exposition. (**A**) Neutralizing antibody titers (inverse) against various SARS-CoV-2 variants. Each data point represents the reciprocal of the neutralizing titer. (**B**) IFN-γ spot increments per 250,000 PBMC in response to different SARS-CoV-2 variants. Each data point represents the increment in IFN-γ spots. Data points are color-coded based on risk level: low-risk (green), intermediate-risk (yellow), and high-risk (red). Horizontal lines indicate mean values, while error bars indicate the standard deviation.

To investigate whether healthcare workers with frequent exposure to COVID-19 patients exhibit elevated titers of neutralizing antibodies against SARS-CoV-2 and its variants, the humoral immune response was analyzed using a cell culture-based neutralization assay. No significant differences in the neutralizing antibody titers were found across the various groups and SARS-CoV-2 variants studied. Interestingly, HCW within the low-risk group exhibited slightly elevated antibody titers against SARS-CoV-2 wild-type D614G, Delta B1.617.2, and Omicron BA.1, surpassing those observed in both the intermediate- and high-risk groups. Participants in the high-risk group exhibited the lowest neutralizing antibody titers against SARS-CoV-2 variants D614G, Delta B1.617.2, and Omicron BA.1. Of note, the median neutralizing titers for Omicron BA.5 were found to be comparable across all groups (Figure 2A). Overall, the neutralizing antibody titers against BA.5 were the lowest (Figure 2A).

The cellular immune response to SARS-CoV-2 wild-type, Delta S.AY, Omicron BA.5, and Omicron BQ1.1 was assessed using an *in-house* IFN-γ ELISpot assay. No significant differences in the cellular immune response were observed among the three groups. The spot increments for all four tested variants were comparable between the low-risk and intermediate-risk groups, whereas volunteers in the high-risk group exhibited the highest spot increments. Notably, the spot increments for the Delta variant were lower compared to the wild-type, Omicron BA.5, and Omicron BQ1.1 (Figure 2B).

### 3.3. Immune Response Is Independent of Vaccination Regimen

**Figure 3:**
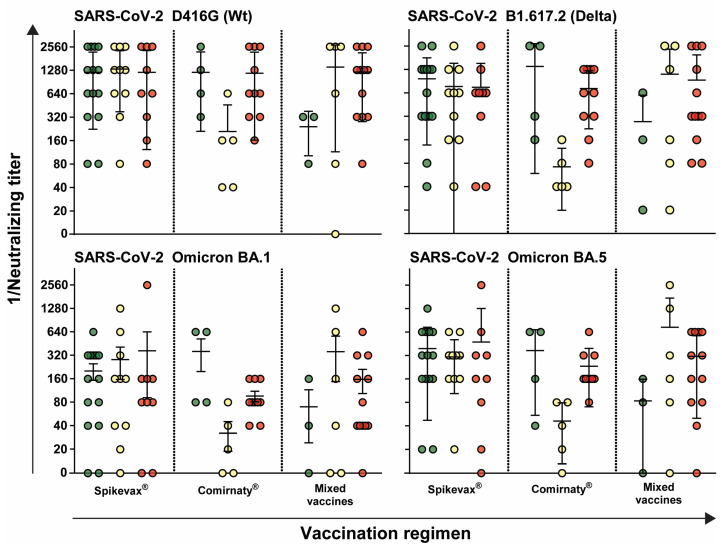
Neutralizing antibody titers against different SARS-CoV-2 variants depending on vaccination regiment. The neutralizing antibody titers of sera from healthcare workers immunized with Spikevax^®^, Comirnaty^®^ or mixed vaccines were determined against the SARS-CoV-2 variants wild-type D416G, Delta B.1.617.2, Omicron BA.1 and Omicron BA.5. Each data point represents the neutralizing titer of an individual, with error bars indicating the standard deviation. Horizontal lines indicate mean values. Data points are color-coded based on risk level: low-risk (green), intermediate-risk (yellow), and high-risk (red).

To conduct a more detailed analysis of whether the vaccine influenced both humoral and cellular immune responses, the data were stratified based on vaccination background. No significant differences between the risk groups were observed for individuals solely vaccinated with Spikevax^®^ (Moderna). There were also no significant differences observed in the antibody titers among individuals vaccinated with Comirnaty^®^ (BioNTech/Pfizer). However, individuals in the low-risk group exhibited the highest antibody titers, whereas those in the intermediate-risk group displayed the lowest antibody titers when vaccinated with Comirnaty^®^. Volunteers in the low-risk group exhibited the lowest antibody titers against all tested variants when vaccinated with different vaccines, although no significant differences were observed. Overall, antibody titers against Omicron variants were lower compared to the wild-type D614G and the Delta variant B1.617.2. Among the individuals who were vaccinated with mixed vaccines, those with intermediate risk showed the highest antibody titers, especially against Omicron BA.1 and Omicron BA.5. (Figure 3).

**Figure 4:**
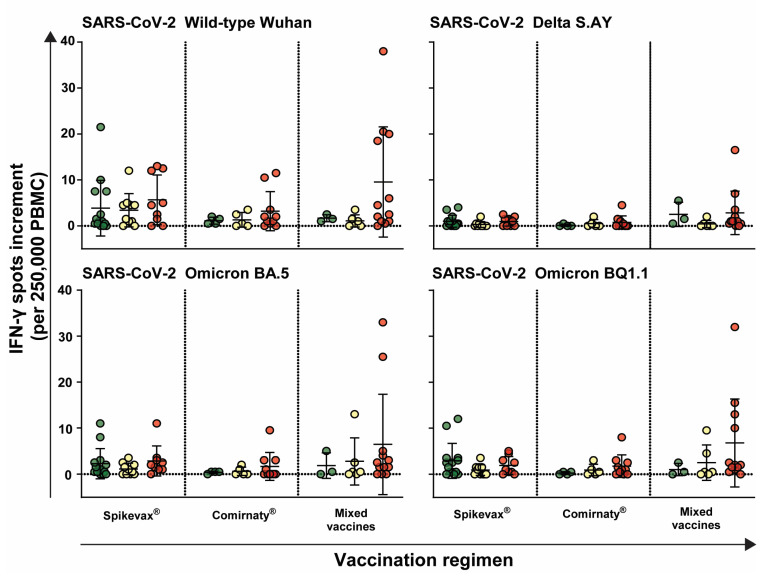
Cellular immune response against different SARS-CoV-2 variants depending on vaccination regiment. The cellular immune responses of healthcare workers immunized with Spikevax^®^, Comirnaty^®^ or mixed vaccines were determined against the SARS-CoV-2 variants wild-type Wuhan, Delta S.AY, Omicron BA.5, and Omicron BQ1.1 as IFN-γ spot increments per 250,000 PBMC. Each data point represents the neutralizing titer of an individual, with error bars indicating the standard deviation. Horizontal lines indicate mean values. Data points are color-coded based on risk level: low-risk (green), intermediate-risk (yellow), and high-risk (red).

For the cellular immune response, no significant differences were observed between the risk groups and vaccination schemes. The spot increments were comparable among individuals vaccinated with Spikevax^®^ (Moderna), regardless of the risk group. However, individuals vaccinated with Comirnaty^®^ (BioNTech/Pfizer) exhibited lower spot increments for all tested variants across all risk groups. The differences in spots increments were not significant between the risk groups vaccinated with different vaccines. Notably, individuals in the high-risk group displayed the highest spot increments, irrespective of the vaccine type and SARS-CoV-2 variant (Figure 4).

### 3.4. HCW Who Were Infected More Recently Exhibited an Increasedimmune Response

**Figure 5:**
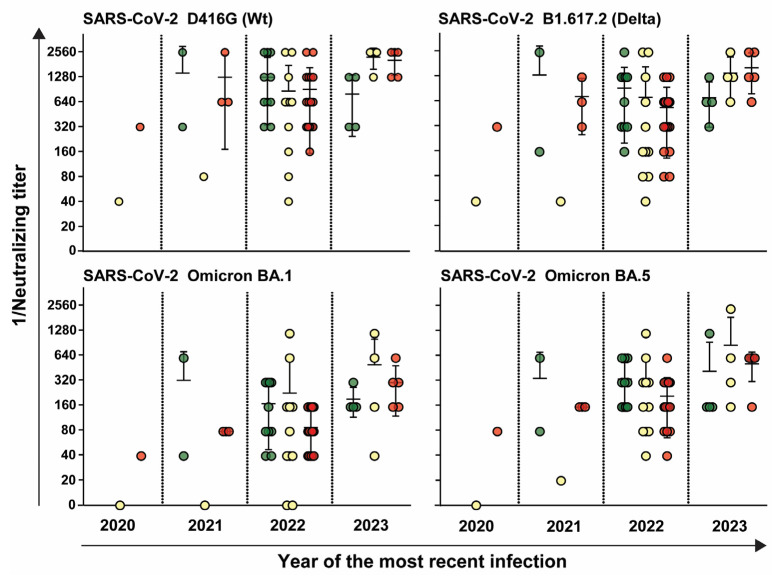
Neutralizing antibody titers against different variants of SARS-CoV-2 stratified by the year of the most recent infection. The neutralizing antibody titers of sera from healthcare workers were determined against the indicated SARS-CoV-2 variants. Each data point represents the neutralizing titer of an individual, with error bars indicating the standard deviation. Horizontal lines indicate mean values. Data points are color-coded based on risk level: low-risk (green), intermediate-risk (yellow), and high-risk (red).

To investigate the impact of recent infection on the humoral and cellular immune response to variants of SARS-CoV-2, HCW were categorized based on the timing of their last SARS-CoV-2 infection. No significant differences were observed among the risk groups in terms of neutralizing antibody titers against wild-type D614G, Delta B1.617.2, Omicron BA.1, and Omicron BA.5. Specifically, antibody titers against wild-type D614G and Delta B1.617.2 were found to be comparable across the risk groups, regardless of the timing of infection. HCW who experienced infection in 2023 exhibited elevated titers for all tested variants of SARS-CoV-2. Interestingly, individuals in the low-risk group who were infected in 2023 displayed slightly lower antibody titers compared to those in the intermediate- or high-risk groups. (Figure 5).

**Figure 6:**
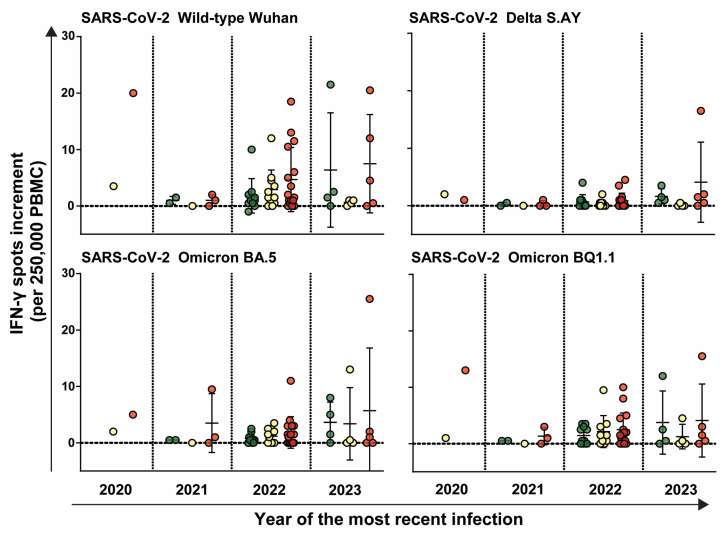
Cellular immune response against different SARS-CoV-2 variants stratified by the year of the most recent infection. The cellular immune response expressed as IFN-γ spot increment (per 250,000 PBMC) to the SARS-CoV-2 variants wild-type Wuhan, Delta S.AY, Omicron BA.5, and Omicron BQ1.1 categorized by the year of the most recent infection. Each data point represents the IFN-γ spot increment for a specific variant of SARS-CoV-2. Data points are color-coded based on risk level: low-risk (green), intermediate-risk (yellow), and high-risk (red). Horizontal lines indicate mean values, while error bars indicate the standard deviation.

Next, we proceeded to examine the cellular immune response of HCW based on the year of their most recent SARS-CoV-2 infection. No significant differences were observed in the cellular immune response between different risk groups and the timing of the most recent SARS-CoV-2 infection. Nevertheless, the increase in spot increments among HCW in the high-risk group was notably higher than among participants in the low- or intermediate-risk group. Additionally, the spot increments for the wild-type of SARS-CoV-2 exceeded that of other variants, irrespective of the timing of infection (Figure 6).

## 4. Discussion

In the present work, we examined the frequency of SARS-CoV-2 infections among HCW with daily contact to COVID-19, those with daily contacts with non-COVID-19 patients, and employees without any contact with patients. Furthermore, we investigated the humoral and cellular immune response of the HCW against SARS-CoV-2 to assess the immunologic status depending on the frequency of patient contact, vaccinations, number of infections, and the time of the most recent preceding infection.

Here, we discovered that three years after the pandemic, the prevalence of SARS-CoV-2 infection and the levels of humoral and cellular immunity were similar among healthcare workers, regardless of their exposure to COVID-19 patients. This finding emphasizes the effectiveness of maintaining high hygiene standards in preventing the transmission of SARS-CoV-2 within healthcare settings. 

There are indications that especially during the Omicron wave, SARS-CoV-2 infections were almost entirely attributable to non-occupational cases contracted outside the hospital [9]. We assume that this factor equally affected all healthcare workers examined in our study. Consequently, the lack of difference in immunity between study groups may also reflect the high transmissibility of the virus and the prevalence of asymptomatic COVID-19 cases. The seroprevalence has shown a steady increase in recent years, with estimates indicating it reached around 50% among the German population at the onset of the study in 2023 [12,21,22]. The actual infection rate will be higher, as asymptomatic infections often go undetected [23]. Furthermore, regular testing of the population no longer occurs, as was the case at the beginning of the pandemic. Studies from the early beginning of the pandemic indicated that the seroprevalence among healthcare workers mirrored that of the general population at the pandemic’s outset [7,8]. Interestingly, 84% of the healthcare workers participating in this study reported at least one SARS-CoV-2 infection, while only 16% had not been infected thus far. Unlike the general population, HCW underwent regular testing for SARS-CoV-2 infection to mitigate potential spread within the hospital. This heightened infection rate likely accounts for the elevated infection rate observed among HCW compared to the general population. 

The approval of effective vaccines played a crucial role in controlling the pandemic and protecting the population. In Germany, COVID-19 vaccination started at the end of December 2020 with the prioritization of high-risk groups, including HCW [23]. By May 2024, over 76% of the German population had been fully vaccinated, with more than 63% having received at least one booster dose [22]. HCW included in this study received vaccinations with Spikevax^®^ (Moderna), Comirnaty^®^ (BioNTech/Pfizer), and/or Vaxzevria (AstraZeneca). While the majority of participants in the low- and intermediate-risk groups received vaccinations solely with Spikevax^®^ (Moderna), those in the high-risk group predominantly received vaccinations with various vaccines. These discrepancies arise from the prioritization of healthcare workers with frequent exposure to COVID-19 patients for vaccination, leading them to receive available vaccines such as the vector-based vaccine from AstraZeneca initially, whereas personnel with less exposure to COVID-19 patients were vaccinated at a later stage. The vaccine efficacy against SARS-CoV-2 infection after two doses of vaccine was reported as 95% for Comirnaty^®^ (BioNTech/Pfizer), 94.1% for Spikevax^®^ (Moderna), and 79% for Vaxzevria (AstraZeneca) [24]. Of note, heterologous prime-boost vaccinations, combining an mRNA vaccine with the vector-based vaccine (Vaxzevria), resulted in markedly higher levels of neutralizing antibody titers compared to vaccinations with either Vaxzevria alone or an mRNA vaccine alone [25]. Overall, we observed no significant differences in neutralizing antibody titers or cellular immune responses, measured as IFN-γ spot increments, between the various vaccination regimens. This can most likely be explained by the fact that the HCW were triple- or quadruple-vaccinated and had already experienced at least one SARS-CoV-2 infection. 

Overall, the neutralizing antibody titers against the Omicron variants BA.1 and BA.5 were lower in all groups compared to the titers against D416G and B1.617.2 (Delta). These findings are consistent with previous studies, which demonstrated a substantial neutralization escape by SARS-CoV-2 Omicron variants, especially BA.5 [26,27].

As a consequence, an increase in breakthrough infections was observed [11]. This is in agreement with another study that showed breakthrough infections can occur despite complete vaccination [28]. However, vaccination still conferred a significant advantage. The vaccines offered effective protection against severe illness [29,30,31] and significantly reduced the infectiousness of vaccinated and/or previously infected individuals compared to those without any history of vaccination or infection [32,33]. 

Interestingly, we observed slightly higher IFN-γ spot increments in HCW exclusively vaccinated with Spikevax^®^ (Moderna), compared to participants vaccinated with Comirnaty^®^ (BioNTech/Pfizer). This discrepancy could be attributed to differences in vaccination dosage. Spikevax^®^ contains 100 µg mRNA per dose [30], whereas Comirnaty^®^ only contains 30 µg mRNA per dose [29]. Participants in the high-risk group exhibited higher spot increments for all variants, regardless of the vaccination scheme. This observation may be attributed to a booster effect resulting from increased exposure to COVID-19 patients.

To further analyze the impact of recent SARS-CoV-2 infections on immune responses, neutralizing antibody titers and IFN-γ spot increments were categorized by the year of the latest infection, stratified by risk group. No significant differences in humoral immune response were observed among the three risk groups based on the year of the latest infection. The highest neutralizing antibody titers and IFN-γ spot increments were observed in HCW with the most recent infections against all tested SARS-CoV-2 variants. The finding correlates with earlier studies that have shown a significant decrease in neutralizing antibody titers and cellular immune responses in vaccinated and convalescent individuals within a few months after booster immunization or infection [34,35,36,37].

The limitations of this study include the cohort size and the disparity in the distribution of the selected risk groups. Specifically, 42% of the participants belonged to the high-risk group, while 29% belonged to either the intermediate-risk group or the low-risk group, respectively. A key limitation of this study is the lack of data on asymptomatic SARS-CoV-2 infections. As screening protocols focused on testing only symptomatic healthcare workers during the Omicron wave in 2023, we were unable to capture the full extent of asymptomatic infections. This likely underestimates the true infection rate, as emerging evidence suggests that Omicron variants may cause a higher proportion of asymptomatic cases compared to prior variants. Future studies should aim to monitor both symptomatic and asymptomatic infections to better characterize transmission risk in healthcare settings. 

## 5. Conclusions

In conclusion, we investigated the prevalence rate of SARS-CoV-2 infection among healthcare workers with varying levels of exposure risk three years after the start of the pandemic. The prevalence of infection and the levels of humoral and cellular immunity were comparable among all groups of healthcare workers, regardless of their exposure to COVID-19 patients. Thus, our epidemiological and immunological data underscore the effectiveness of high hygiene standards in preventing SARS-CoV-2 in healthcare settings. Further comprehensive epidemiological studies are necessary to elucidate the determinants of both cellular and humoral immunity, as well as to evaluate the impact of relevant predictors on the risk of SARS-CoV-2 infection. These predictors should include virus variants, occupational risk factors, vaccination status, number of previous infections, age, and sex, among others.

## Figures and Tables

**Table 1:** Distribution of healthcare workers by occupational risk of COVID-19 infection.

	Low-Risk	Intermediate-Risk	High-Risk	Overall
**Total number**	21	21	31	73
**Gender (m/f)**	5/16	2/19	12/19	19/54
**Median age (range)**	34 (22–62)	34 (23–62)	39 (23–70)	37 (22–70)
**Nurse**	0	2 (10%)	14 (45%)	16
**Physician**	1 (5%)	4 (19%)	14 (45%)	19
**Lab assistant**	13 (62%)	0	0	13
**Other**	7 (33%)	15 (71%)	3 (10%)	25

## Data Availability

Data are available on request from the corresponding author.
